# Genomic correlation, shared loci, and causal relationship between obesity and polycystic ovary syndrome: a large-scale genome-wide cross-trait analysis

**DOI:** 10.1186/s12916-022-02238-y

**Published:** 2022-02-11

**Authors:** Qianwen Liu, Zhaozhong Zhu, Peter Kraft, Qiaolin Deng, Elisabet Stener-Victorin, Xia Jiang

**Affiliations:** 1 Department of Clinical Neuroscience, Center for Molecular Medicine, Karolinska Institutet, Solna, Stockholm Sweden; 2 Department of Emergency Medicine, Massachusetts General Hospital, Harvard Medical School, Boston, MA USA; 3 Department of Biostatistics, Harvard T.H. Chan School of Public Health, Boston, MA USA; 4 Department of Epidemiology, Harvard T.H. Chan School of Public Health, Boston, MA USA; 5 Department of Physiology and Pharmacology, Karolinska Institutet, Stockholm, Sweden

**Keywords:** Polycystic ovary syndrome, Obesity, Body mass index, Fat distribution, Genome-wide cross-trait analysis

## Abstract

**Background:**

The comorbidity between polycystic ovary syndrome (PCOS) and obesity has long been observed in clinical settings, but their shared genetic basis remains unclear.

**Methods:**

Leveraging summary statistics of large-scale GWAS(s) conducted in European-ancestry populations on body mass index (adult BMI, *N*_female_=434,794; childhood BMI, *N*=39,620), waist-to-hip ratio (WHR, *N*_female_=381,152), WHR adjusted for BMI (WHR_adj_BMI, *N*_female_=379,501), and PCOS (*N*_case_=10,074, *N*_control_=103,164), we performed a large-scale genome-wide cross-trait analysis to quantify overall and local genetic correlation, to identify shared loci, and to infer causal relationship.

**Results:**

We found positive genetic correlations between PCOS and adult BMI (*r*_*g*_=0.47, *P*=2.19×10^−16^), childhood BMI (*r*_*g*_=0.31, *P*=6.72×10^−5^), and WHR (*r*_*g*_=0.32, *P*=1.34×10^−10^), all withstanding Bonferroni correction. A suggestive significant genetic correlation was found between PCOS and WHR_adj_BMI (*r*_*g*_=0.09, *P*=0.04). Partitioning the whole genome into 1703 nearly independent regions, we observed a significant local genetic correlation for adult BMI and PCOS at chromosome 18: 57630483–59020751. We identified 16 shared loci underlying PCOS and obesity-related traits via cross-trait meta-analysis including 9 loci shared between BMI and PCOS (adult BMI and PCOS: 5 loci; childhood BMI and PCOS: 4 loci), 6 loci shared between WHR and PCOS, and 5 loci shared between WHR_adj_BMI and PCOS. Mendelian randomization (MR) supported the causal roles of both adult BMI (OR=2.92, 95% CI=2.33–3.67) and childhood BMI (OR=2.76, 95% CI=2.09–3.66) in PCOS, but not WHR (OR=1.19, 95% CI=0.93–1.52) or WHR_adj_BMI (OR=1.03, 95% CI=0.87–1.22). Genetic predisposition to PCOS did not seem to influence the risk of obesity-related traits.

**Conclusions:**

Our cross-trait analysis suggests a shared genetic basis underlying obesity and PCOS and provides novel insights into the biological mechanisms underlying these complex traits. Our work informs public health intervention by confirming the important role of weight management in PCOS prevention.

**Supplementary Information:**

The online version contains supplementary material available at 10.1186/s12916-022-02238-y.

## Background

Polycystic ovary syndrome (PCOS) is the most common endocrine disorder affecting women of childbearing age, characterized by reproductive dysfunction including hyperandrogenism, menstrual and/or ovulatory irregularity together with subfertility, and metabolic dysfunction including hyperinsulinemia, insulin resistance, and type 2 diabetes [1, 2]. More than 50% of women with PCOS are either overweight or obese which further worsens all symptoms [3]. Indeed, epidemiological studies have observed a significant association between body mass index (BMI) and features of PCOS at all ages [4]. Clinically, even a modest weight loss (~ 5%) leads to meaningful improvements in the reproductive, hyperandrogenic, and metabolic features of PCOS [5], highlighting a biological link underlying obesity and PCOS.

The development of obesity and PCOS involves strong genetic components evidenced by recent discoveries from large-scale genome-wide associations studies (GWAS). These genetic data enable the utilization of a compiled analytical strategy—genome-wide cross-trait analysis—to determine shared and distinct genetic architecture which can provide better understandings and novel insights into disease mechanisms [6]. Such analysis features several analytic aspects: genetic correlation analysis to estimate overall and local genetic correlation, cross-trait meta-analysis to identify shared loci, and Mendelian randomizations (MR) to make causal inferences. Nevertheless, these advanced statistical genetics approaches have not been routinely applied to examine the genetic contribution to the epidemiologic associations between PCOS and its most common comorbidity, obesity [7, 8]. Despite three MR studies [7, 9, 10] have been conducted to explore the role of adult BMI in PCOS, these studies used a small number of index SNPs (< 100 instruments vs. ~ 300 female-specific BMI instruments currently identified by GWAS [11]); lacked sensitivity analyses to verify model assumptions; and lacked sex-specific analysis, i.e., using genetic data derived from a sex-mixed population instead of using female-specific data for a gynecological outcome PCOS.

In addition to the degree of adiposity, location and distribution of fat accumulation are informative predictors for obesity sequelae. For example, abdominal visceral fat, a known contributor to metabolic dysfunction including insulin resistance and abnormal adipokine and fatty acid release [12], is important for PCOS. Furthermore, early life weight pattern also influences obesity and metabolic alterations later on [13]. However, to the best of our knowledge, genetic analysis has rarely been conducted to examine the role of fat distribution or childhood BMI in PCOS [7].

**Fig. 1:**
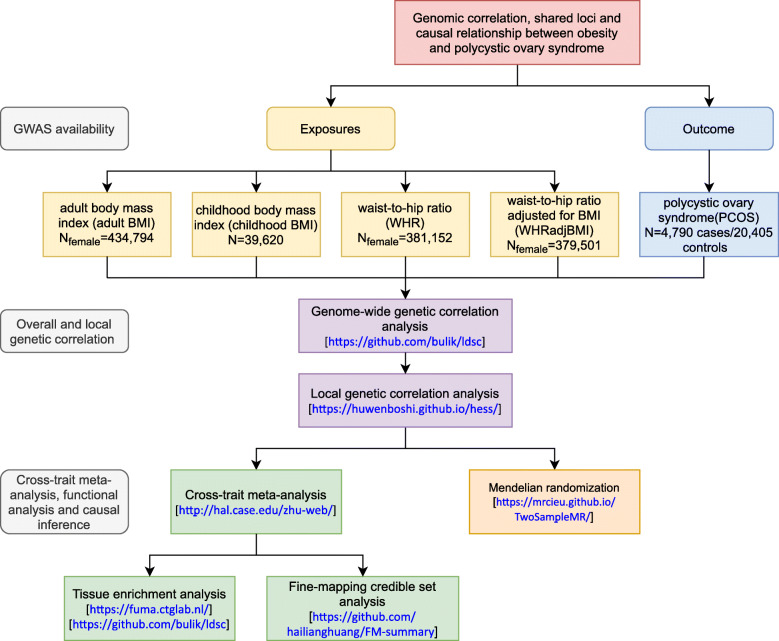
Overall study design. GWAS summary statistics on PCOS and 4 obesity-related traits (adult BMI, childhood BMI, WHR, and WHR_adj_BMI) were retrieved. First, we quantified the genome-wide and local genetic correlation between PCOS and obesity-related traits. Next, we identified shared loci contributing to PCOS and obesity-related traits, and further, conducted tissue enrichment analysis, and constructed 99% credible sets of causal variants for these loci. Finally, we conducted a bidirectional Mendelian randomization analysis to infer causality between obesity-related traits and PCOS

Therefore, we aim to extend previous findings by providing a systematic evaluation of the relationship between obesity-related traits and PCOS, leveraging the hitherto largest GWAS summary statistics conducted for each trait. We examined the role of BMI (childhood (before age 10) (*N*=39,620) [14] and adult [11]), waist-to-hip ratio [15] (WHR, adult), and waist-to-hip ratio adjusted for BMI [15] (WHR_adj_BMI, adult) (all adult measures were restricted to female participants, *N*_Female_=~ 400,000) in the development of PCOS (*N*_PCOS_=10,074; *N*_control_=103,164) [7], performing analyses to quantify overall and local genetic correlations, to identify shared loci and to infer causal relationships. A conceptual framework is shown in Fig. 1.

## Methods

We carried out the current study by leveraging large-scale GWAS summary statistics and novel statistical genetics approaches. We included female-specific genetic data of adult obesity-related traits to best match with a female-specific outcome PCOS. However, sex-specific data for childhood BMI were unavailable. To reduce potential bias from population stratification, all genetic data were restricted to European population.

### Obesity GWAS(s)

For *adult obesity*, the hitherto largest GWAS was conducted meta-analyzing data from UK Biobank and GIANT consortium totaling ~ 700,000 individuals of European ancestry, among which 434,794 female participants with information available for BMI, 381,152 for WHR, and 315,284 for WHR_adj_BMI [11, 15]. In each participating study, SNPs were imputed to the Haplotype Reference Consortium (HRC) reference panel and filtered by imputation quality score > 0.30, call rate > 0.95, minor allele frequency > 0.0001 and *P* value for Hardy-Weinberg equilibrium > 10^−6^. After quality control, genome-wide association testing was performed using a linear mixed model adjusting for age, recruitment center, genotyping batches, and principal components. A fixed-effect inverse-variance-weighted meta-analysis was conducted to combine effect sizes across studies. To identify independent top-associated SNPs, a PLINK clumping algorithm of *P*< 1×10^− 9^ and an LD window of ±5 Mb (*r*^2^ > 0.05) were first used to obtain LD-based clumps [16], followed by a proximal conditional and joint testing to identify primary and secondary signals within each of the clumping-based loci.

For *childhood BMI*, the hitherto largest GWAS was conducted at the latest time point between 2 and 10 years old among 61,111 children of European ancestry [14]. In the discovery stage, data from 26 studies (*N*_discovery_ = 39,620) imputed to the 1000 Genomes Project or the HRC were analyzed using a fixed-effect inverse variance-weighted meta-analysis. Top associated SNPs identified in the discovery stage at a *P*-threshold of 5×10^−6^ were taken forward for replication in 15 cohorts (*N*_replication_ = 21,491) and results of the two stages were combined using meta-analysis. To identify independent top-associated SNPs, Genome-wide Complex Trait Analysis based on summary statistics with LD estimation from the Generation R Study as a reference sample was applied. Top associated SNPs in the combined meta-analysis reaching a stringent *P*-threshold of 5×10^−8^ were identified.

From these GWAS(s), we extracted the effect size and relevant information of instrumental variables (IVs) identified in women (281 BMI-associated index SNPs, 203 WHR-associated index SNPs, and 266 WHR_adj_BMI-associated index SNPs, Additional file 1: Tables S1-3) and children (25 childhood BMI-associated index SNPs, Additional file 1: Table S4). We also accessed and downloaded the full set GWAS summary statistics.

### PCOS GWAS

The hitherto largest GWAS of PCOS was conducted based on international collaborations in 10,074 PCOS cases and 103,164 controls of European ancestry [7]. Data from 7 cohorts were imputed to the 1000 Genomes Project or HapMap2. Diagnosis of PCOS was based on the NIH (2540 cases/15,020 controls) or Rotterdam criteria (2669 cases/17,035 controls), or by self-reported diagnosis (5184 cases/82,759 controls, 23andMe). A fixed-effect inverse-variance weighted meta-GWAS was performed adjusting for age. To identify independent top-associated SNPs, a fixed-effect inverse-weighted-variance meta-analysis was applied to obtain the per-variant estimates from summary statistics of contributing studies. This GWAS identified 14 PCOS-associated variants reaching a P-threshold of 5×10^−8^. From PCOS GWAS, we extracted IV_obesity_-PCOS associations and relevant information. We also downloaded the full set GWAS summary statistics of PCOS in which data from 23andMe were excluded (due to data availability) (Additional file 1: Table. S5).

A table summarizing the information of all included GWASs is shown in Additional file 1: Table. S6 and a table summarizing all participating studies is shown Additional file 1: Table. S7. For all analyses, the human reference genome build 37 (or hg19) was used.

### Statistical analysis

#### Genetic correlation analysis

Genome-wide genetic correlations (*r*_*g*_) quantifies the average sharing of genetic effect between two traits unaffected by environmental confounders. The estimate ranges from − 1 to 1, with − 1 indicating a perfect negative genetic correlation and 1 indicating a perfect positive genetic correlation. It can be quantified using GWAS summary data through an algorithm implemented in software linkage-disequilibrium score regression (LDSC) [17, 18] also described below:

$$ E\left[{\upbeta}_j{\upgamma}_j\right]=\frac{\sqrt{N_1{N}_2}{r}_g}{M}{l}_j+\frac{N_sr}{\sqrt{N_1{N}_2}} $$

where *β*_*j*_ and *γ*_*j*_ are the effect sizes of SNP *j* on traits 1 and 2, *r*_*g*_ is the genetic covariance, *M* is number of SNPs, N_1_ and N_2_ are the sample sizes for traits 1 and 2, *N*_*s*_ is the number of overlapping samples, *r* is the phenotypic correlation in overlapping samples, and *l*_*j*_ is the linkage disequilibrium score. We used pre-computed LD scores obtained from ~ 1.2 million common SNPs in European ancestry represented in the HapMap3 reference panel, commonly recognized as of high imputation quality.

Genome-wide genetic correlations estimated by LDSC quantify the genome-wide contribution of genetic variation to the correlation between two traits. It is possible that even though two traits show negligible genome-wide genetic correlation, genetic variations localized at a specific genomic region contribute to the correlation between two traits. We, therefore, measured the pairwise local genetic correlations between each of the obesity-related traits and PCOS using ρ-HESS, an algorithm that partitions the genome-wide genetic sharing across 1703 nearly independent linkage disequilibrium (LD) regions of 1.5 Mb and precisely quantifies the genetic correlation between pairs of traits due to genetic variation restricted to these genomic regions.

#### Cross-trait meta-analysis

Cross-phenotype association analysis (CPASSOC) integrates GWAS summary statistics from multiple correlated traits to detect evidence for variants associated with multiple traits across studies while controlling population structure and cryptic relatedness [19]. CPASSOC provides two test statistics, S_Hom_ and S_Het._ S_Hom_ is based on the fixed-effect meta-analysis method and is the most powerful when genetic effect sizes are homogenous, which is unlikely to be true especially when multiple traits are analyzed. S_Het_ is an extension of S_Hom_ with improved power that allows for heterogeneous effects of a trait from different study designs, environmental factors, or populations, as well as heterogeneous effects for different phenotypes, which is more common in practice. We used pairwise S_Het_ to combine summary statistics for each of the obesity-related traits with PCOS. We applied PLINK [16] clumping function parameters: --clump-p1 5e-8 --clump-p2 1e-5 --clump-r2 0.2 --clump-kb 500) to obtain independent SNPs. Significant SNPs were defined as variants with *P*_single trait_< 1×10^−3^ (in each single trait) and *P*_CPASSOC_< 5×10^−8^ (in cross-traits). We used Ensembl Variant Effect Predictor (VEP) for detailed functional annotation for the variants identified by CPASSOC.

#### Fine-mapping credible set analysis

Index SNP does not necessarily represent causal variants. We further identified a 99% credible set of causal variants through a simplified Bayesian fine-mapping method named FM-summary (https://github.com/hailianghuang/FM-summary) [20]. Briefly, for each of the 16 shared loci identified as significant by the cross-trait meta-analysis, we extracted variants within 500 kb around the index SNP, which were used as input for FM-summary. FM-summary then set a flat prior and produced a posterior inclusion probability (PIP) of a true trait/disease association for each variant using the steepest descent approximation. A 99% credible set is equivalent to ranking SNPs from largest to smallest PIPs and taking the cumulative sum of PIPs until it is at least 99%. Details of the method were described elsewhere [21, 22].

#### Functional annotation and tissue enrichment analysis

To gain putative biological insights into the shared variants identified between obesity and PCOS, we performed GTEx tissue enrichment analysis including all genes in clumping regions for each trait identified by CPASSOC using software functional mapping and annotation (FUMA), the GENE2FUNC process with 54 tissue types from GTEx (version 8). FUMA obtained differentially expressed gene (DEG) sets for each tissue type by using the normalized expression (zero mean of log2(RPKM+1)) and conducting two-sided t-tests per gene per tissue. Genes were defined as DEG set in a specific tissue if Bonferroni corrected P-value<0.05 and had an absolute log-fold change ≥ 0.58 (background DEGs). Genes identified by CPASSOC were tested against those background DEG sets by hypergeometric tests to examine if they were overrepresented in DEG sets in specific tissue types, thus, identifying the most relevant tissue types.

To identify tissue and cell-type-specific enrichment of SNPs, we used LD score regression applied to specifically expressed genes (LDSC-SEG) [23] which tests for enrichment for per-SNP heritability. Pre-computed annotations constructed from epigenomics data from Roadmap Epigenomics Project [24] (DNase hypersensitivity, H3K27ac, H3K4me3, H3K4me1, H3K9ac, and/or H3K36me3 chromatin marks) were used in our analysis. The 396 cell-type annotations were further divided into 9 groups (adipose, central nervous system (CNS), digestive system, cardiovascular, musculoskeletal and connective tissue, immune and blood, liver, pancreas, and others). To correct for multiple comparison, a Bonferroni-corrected *P*-threshold (0.05/396) was used.

#### Mendelian randomization analysis

To test for the causal relationship between each of the obesity-related traits and PCOS, we conducted a two-sample MR. We applied an inverse-variance weighted (IVW) approach [25] as our primary MR analysis, an MR-Egger regression [26], and a weighted-median estimator approach [27] to examine the robustness of our findings under relaxed model assumptions.

We performed important sensitivity analyses to validify MR model assumptions. We excluded palindromic IVs with strand ambiguity (A/T or G/C SNPs with the same pair of letters on the forward and reverse strands, introducing ambiguity into strand identity) and pleiotropic SNPs (SNPs associated with potential confounding traits other than exposure and outcome of interest) according to GWAS catalog (https://www.ebi.ac.uk/gwas/, accessed on 03/18/2021). We conducted a leave-one-out analysis where we removed one IV each time and performed IVW using the remaining IVs to identify outlying instruments. We also examined a scenario through MR-Clust [28] where several distinct causal mechanisms may underlie the obesity-PCOS relationship (i.e., a risk factor influences outcome with different magnitudes and direction of causal effect). MR-Clust divides IVs into distinct clusters such that all variants in the cluster have similar causal estimates. To examine the causal effect of the genetic predisposition to PCOS on obesity, we finally performed a bidirectional MR analysis where instruments for outcomes were used to evaluate whether the “outcome” (here, PCOS) caused the “exposure” (here, obesity-related exposures). The 14 PCOS-associated independent loci with genome-wide significance were included as IVs in our reverse direction MR and their effects were extracted from the respective obesity GWAS(s).

## Results

### Genetic correlations between obesity-related traits and PCOS

We first estimated the overall genetic correlation between obesity-related traits and PCOS using cross-trait LDSC. After correcting for multiple testing (*P*< 0.05/4), we found a strong genetic correlation between BMI and PCOS (adult BMI: *r*_*g*_=0.47, *P*=2.19×10^−16^; childhood BMI: *r*_*g*_=0.31, *P*=6.72×10^−5^) (Table 1). A significant result was also observed for WHR and PCOS (*r*_*g*_=0.32, *P*=1.34×10^−10^). Given the complex interplay between BMI and WHR [29], we continued to investigate WHR_ad_jBMI, a residual component of WHR in which the effect of BMI was removed. Perhaps not surprisingly, when the effect of BMI was removed from WHR (WHR_adj_BMI), the prior positive genetic correlation between PCOS and WHR was attenuated to null (WHR_adj_BMI: *r*_*g*_=0.09, *P*=0.04).

**Table 1:** Genome-wide genetic correlation between PCOS and obesity-related traits

Trait 1	Trait 2	*r*_*g*_	*r*_*g*__SE	*P* value
PCOS	BMI	0.4694	0.0572	2.19×10^−16^
PCOS	WHR	0.3198	0.0498	1.34×10^−10^
PCOS	WHR_adj_BMI	0.0931	0.0447	0.0371
PCOS	CBMI	0.3109	0.0078	6.72×10^−5^

*r*_*g*_, genetic correlation; *SE*, standard error; *BMI*, adult body mass index; *PCOS*, polycystic ovary syndrome; *WHR*; waist-to-hip ratio; *WHR*_*adj*_*BMI*, waist-to-hip ratio adjusted for body mass index; *CBMI*, childhood body mass index

**Fig. 2:**
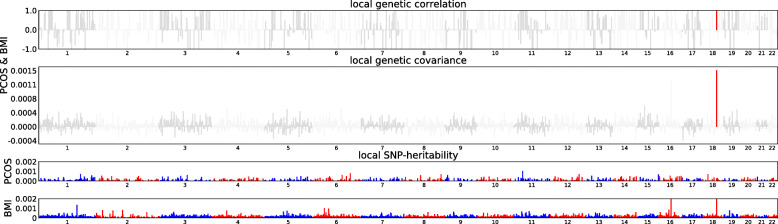
Local genetic correlation, genetic covariance, and SNP heritability between adult BMI and PCOS. Colored bars represent loci that have significant local genetic correlation and covariance after multiple testing adjustment. BMI, adult body mass index; PCOS, polycystic ovary syndrome

Motivated by these findings, we further explored the local genetic correlation using ρ-HESS. As shown in Fig. 2 and Additional file 2: Fig. S1, after correcting for multiple testing (*P*< 0.05/1703), a significant local genetic correlation was only observed for adult BMI and PCOS at chr18: 57630483–59020751, a genetic region harboring *MC4R*, a locus previously reported to be associated with adult BMI, childhood BMI and obesity in PCOS [14, 30–33].

### Cross-trait meta-analysis of obesity-related traits and PCOS

To identify individual SNPs affecting both obesity-related traits and PCOS, we next conducted a pairwise CPASSOC analysis. As shown in Table 2, in total we identified 9 independent loci shared between BMI and PCOS (adult: 5 loci, rs10938397, rs705696, rs1569979, rs10142183, and rs11672660; childhood: 4 loci, rs12641981, rs10987375, rs8050136, and rs7228430), 6 independent loci shared between WHR and PCOS (rs3767846, rs13032835, rs188488867, rs7774737, rs1421085, and rs310011) and 5 independent loci shared between WHR_adj_BMI and PCOS (rs3767846, rs13032835, rs7774737, rs11057429, and rs310011) (all these SNPs fulfilled *P*_single trait_< 1×10^−3^ and *P*_CPASSOC_< 5×10^−8^). Notably, none of these 16 SNPs identified by CPASSOC were previously reported to be associated with PCOS at genome-wide significance (0 out of 16 SNPs), while most of them were associated with obesity-related traits (14 out of 16 SNPs). For adult BMI and PCOS, the most significant shared SNP was rs10938397, (*P*_CPASSOC_=1.25×10^−49^) located at an intergenic region. The second most significant shared SNP (rs11672660, *P*_CPASSOC_=1.87×10^−32^) was located near *GIPR*, a gene associated with BMI and glucose tolerance [34, 35], and *MIR642A*, a gene involved in post-transcriptional regulation of gene expression. Notably, among the 5 adult BMI-PCOS shared SNPs, rs705696 was near *ERBB3*, a locus known to be associated with PCOS. For childhood BMI and PCOS, the most significant shared SNP (rs8050136, *P*_CPASSOC_=1.56×10^−21^) was near *FTO*, a locus known to affect BMI and the predisposition to childhood and adult obesity [36, 37]. Similarly, the most significant shared SNP for WHR and PCOS (rs1421085, *P*_CPASSOC_=1.97×10^−51^) was also near the *FTO* locus. Among the 6 variants associated with both WHR and PCOS, 4 were also found to be shared between WHR_adj_BMI and PCOS (rs3767846, rs13032835, rs7774737, and rs310011). For example, rs3767846 was near *PROX1*, a locus associated with fasting glucose-related traits [38]. The most significant variant shared by WHR_adj_BMI and PCOS (rs13032835, *P*_CPASSOC_=2.74×10^−30^) was located near genes *SLC38A11* and *RNA5SP111*. *SLC38A11* encodes a protein that belongs to the solute carrier family and has a role in sodium and amino acid transportation [39], and *RNA5SP111* is a 5S ribosomal pseudogene [40]. Detailed annotations of each variant are shown in Additional file 1: Table. S8. Of note, some of the CPASSOC-identified significant SNPs were not mapped to any genes (7 out of 16).

**Table 2:** Cross-trait meta-analysis between obesity-related traits and PCOS (*P*-CPASSOC < 5× 10^−8^, single trait *P* value < 1× 10^−3^)

SNP	CHR	BP	Alleles (E/A)	BETA (SD)		*P*-trait	*P*-PCOS	*P*-CPASSOC	Genomic coordinates	Genes within clumping area
				trait	PCOS					
BMI and PCOS										
rs10938397	4	45182527	A/G	− 0.03	− 0.10	1.66×10^−45^	8.90×10^−04^	1.25× 10^−49^	45068929–45186832	
rs705696	12	56480648	A/G	− 0.02	0.13	4.36×10^−09^	1.10× 10^−04^	6.39× 10^−10^	56364321–56564811	CDK2, ERBB3, ESYT1, IKZF4, MYL6, MYL6B, PA2G4, RAB5B, RPL41, RPS26, SMARCC2, SUOX, ZC3H10
rs1569979	14	29681294	A/G	0.02	0.12	1.56×10^−17^	9.80× 10^−04^	1.58× 10^−18^	29549249–29702590	
rs10142183	14	29723729	C/G	0.02	0.17	1.34× 10^−12^	4.00× 10^−05^	8.00× 10^−14^	29662355–29856951	
rs11672660	19	46180184	T/C	− 0.03	− 0.13	5.93×10^−31^	4.70× 10^−04^	1.87× 10^−32^	46172278–46214297	FBXO46, GIPR, MIR642A, MIR642B, QPCTL, SNRPD2
WHR and PCOS										
rs3767846	1	214175126	A/G	0.02	0.13	5.69×10^−11^	5.50× 10^−04^	6.19× 10^−12^	214173840–214192133	PROX1
rs13032835	2	165748563	A/T	− 0.02	− 0.13	2.42× 10^−18^	5.00× 10^−05^	3.59× 10^−20^	165668018–165828042	COBLL1, LOC101929633, SLC38A11
rs188488867	2	219784256	T/C	− 0.04	0.31	9.03×10^−08^	3.00× 10^−04^	1.35× 10^−08^	219755011–219849633	CDK5R2, FEV, LINC00608, LOC101928537, WNT10A
rs7774737	6	81145901	C/G	− 0.04	− 0.38	3.22×10^−10^	5.10× 10^−06^	7.92× 10^−11^	80846547–81597411	BCKDHB
rs1421085	16	53800954	T/C	− 0.03	− 0.13	6.44× 10^−47^	2.00× 10^−05^	1.97× 10^−51^	53797565–53848561	FTO
rs310011	16	81471654	A/G	0.02	0.12	1.84×10^−11^	1.90× 10^−04^	2.30× 10^−12^	81449060–81471654	
WHR_adj_BMI and PCOS										
rs3767846	1	214175126	A/G	0.02	0.13	2.12×10^−08^	5.50× 10^−04^	9.99× 10^−09^	214173840–214189783	PROX1
rs13032835	2	165748563	A/T	− 0.03	− 0.13	5.10×10^−27^	5.00× 10^−05^	2.74× 10^−30^	165668018–165828042	COBLL1, LOC101929633, SLC38A11
rs7774737	6	81145901	C/G	− 0.03	− 0.38	4.02×10^−08^	5.10× 10^−06^	8.49× 10^−09^	81145901–81196080	
rs11057429	12	124529194	C/G	− 0.09	0.52	2.74× 10^−09^	4.20× 10^−04^	4.87× 10^−10^	124529194–124529194	ZNF664-FAM101A
rs310011	16	81471654	A/G	0.02	0.12	1.20×10^−14^	1.90× 10^−04^	3.06× 10^−16^	81449060–81471654	
CBMI and PCOS										
rs12641981	4	45179883	T/C	0.04	0.10	4.19×10^−08^	8.90× 10^−04^	8.24× 10^−10^	45175691–45187622	
rs10987375	9	129392365	T/C	0.04	0.14	2.37×10^−06^	3.60× 10^−04^	2.51× 10^−08^	129370576–129397014	LMX1B
rs8050136	16	53816275	A/C	0.07	0.15	2.24×10^−17^	4.50× 10^−06^	1.56× 10^−21^	53797908–53848561	FTO
rs7228430	18	57733925	A/G	0.05	0.12	5.02×10^−09^	9.50× 10^−04^	5.50× 10^−11^	57730096–57914679	

*SNP*, single nucleotide polymorphisms; *CHR*, chromosome; *BP*, physical position of SNP (base-pairs); *E*, effect allele; *A*, alternative allele; *BETA*, effect allele beta coefficient; *P-trait*, *P* value for each obesity-related trait; *P-PCOS*, *P* value for PCOS; *P-CPASSOC*, *P* value for cross-phenotype association; *BMI*, adult body mass index; *PCOS*, polycystic ovary syndrome; *WHR*, waist-to-hip ratio; *WHR*_*adj*_*BMI*, waist-to-hip ratio adjusted for body mass index; *CBMI*, childhood body mass index

### Functional annotation and tissue enrichment analysis

We attempted to understand the underlying biological mechanisms by identifying relevant tissues using FUMA. As shown in Additional file 2: Fig. S2, for the expression of genes shared by adult BMI and PCOS, although failing to pass multiple correction, we observed enrichment in coronary artery tissue, digestive tract tissues including salivary gland, stomach, and colon, as well as both visceral omentum and subcutaneous adipose tissues. For WHR-PCOS and WHR_adj_BMI-PCOS shared genes, stomach showed the most significant enrichment although notwithstanding multiple correction. No significant tissue enrichment was identified for childhood BMI and PCOS. For LDSC-SEG enrichment analysis, as shown in Additional file 2: Fig. S3, despite significant enrichment of SNPs in adipose, brain, muscle tissues identified for obesity-related traits, none of the tissue/cell-type-specific enrichment withstood multiple correction (*P*< 0.05/396) for PCOS.

### Fine-mapping credible set analysis

For each of the 16 shared loci identified as significant by the cross-trait meta-analysis, we identified a 99% credible set of causal variants. Lists of credible set SNPs in each shared locus for obesity-related traits and PCOS from fine mapping are shown in Additional file 1: Table. S9. We identified 39 SNPs in the 99% credible set for adult BMI and PCOS, 122 SNPs for childhood BMI and PCOS, 40 SNPs for WHR and PCOS, and 27 SNPs for WHR_adj_BMI and PCOS providing candidates for downstream experimental analysis.

### Mendelian randomization analysis of obesity-related traits and PCOS

**Fig. 3:**
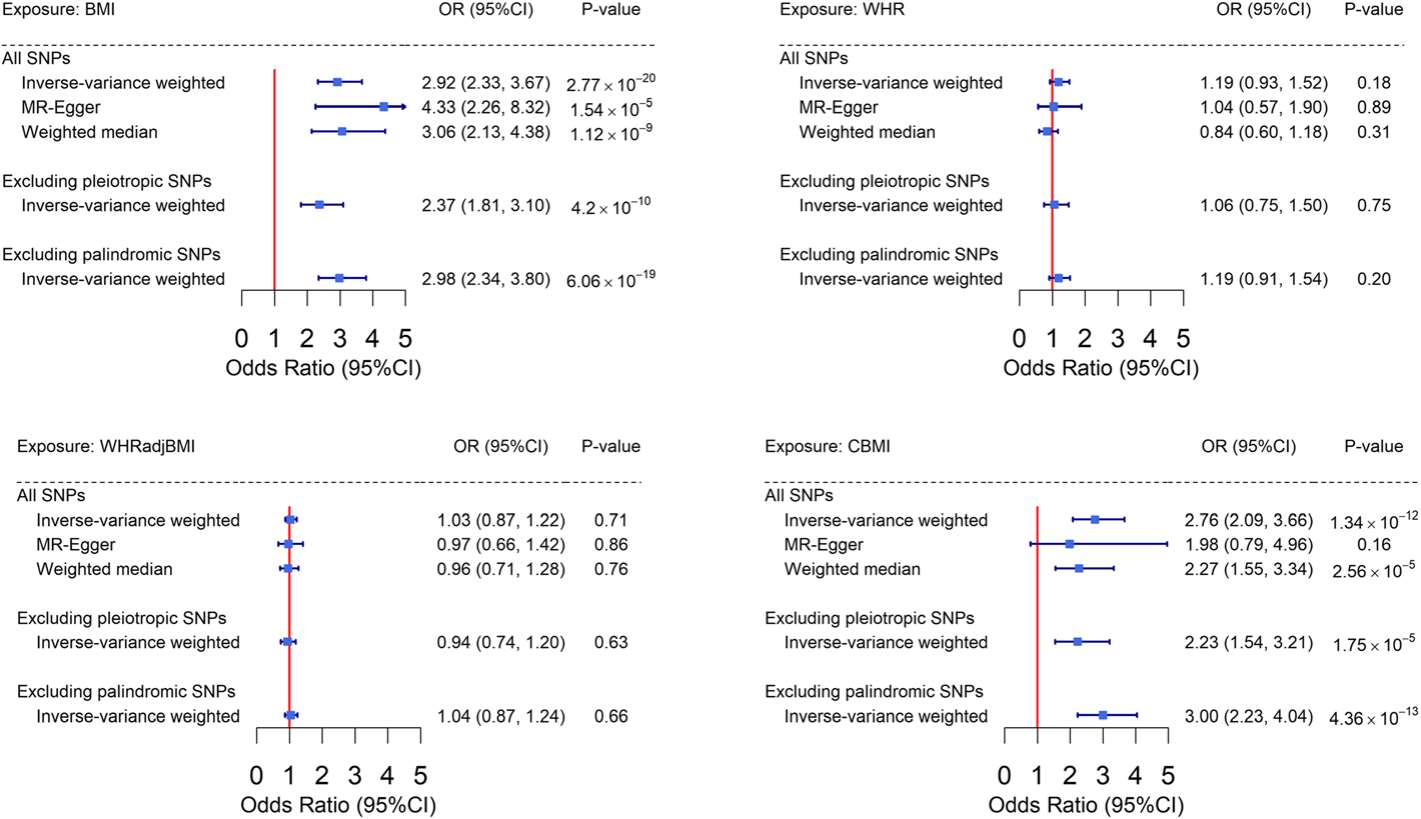
Estimates of causal effect sizes for genetically predicted obesity-related traits on PCOS using all genome-wide significant SNPs, excluding pleiotropic SNPs and excluding palindromic SNPs. Inverse-variance weighted approach was used as the primary analysis, MR-Egger and weighted median approaches were used as sensitivity analysis. BMI, adult body mass index; PCOS, polycystic ovary syndrome; WHR, waist-to-hip ratio; WHR_adj_BMI, waist-to-hip ratio adjusted for body mass index; CBMI, childhood body mass index

Finally, we conducted a bidirectional two-sample Mendelian randomization analysis to test for the causal relationship between obesity-related traits and PCOS. As shown in Figure 3, we observed an almost threefold increased risk of PCOS per-SD increment (4.8 kg/m^2^) in adult BMI (IVW OR=2.92, 95% CI=2.33–3.67) using 278 female-specific IVs. The effect did not alter using MR-Egger (OR=4.33, 95% CI=2.26–8.32) or weighted median (OR=3.06, 95% CI=2.14–4.36) approach. We did not observe any sign of horizontal pleiotropy (*P* for MR-Egger intercept=0.21). Sensitivity analysis removing palindromic SNPs or pleiotropic SNPs (Fig. 3, Supplementary Tables 1-4) revealed similar findings.

Consistent with results from the adult population, we observed a strong causal link with PCOS using 25 childhood BMI-associated IVs (IVW OR=2.76, 95% CI=2.09–3.66)—a less than 10% number of IVs compared to adult BMI. Unfortunately, and perhaps not surprisingly, we were underpowered to perform sensitivity analyses for this trait due to the limited number of IVs. Directional consistent results were observed in MR-Egger analysis although the significance was attenuated to null (MR-Egger OR=1.98, 95% CI=0.79–4.96) (Fig. 3).

On the contrary, we did not find any significant association between genetically predicted WHR and risk of PCOS (IVW OR=1.19, 95% CI=0.93–1.52; MR-Egger OR=1.04, 95% CI=0.57–1.90; weighted median OR=0.84, 95% CI=0.6–1.18). Our results imply a causal role of BMI but not WHR in the development of PCOS. We anticipate observing a null effect of WHR_adj_BMI with PCOS after eliminating the positive association of BMI from the null association of WHR. As expected, we did not identify any increased risk of PCOS with WHR_adj_BMI with all effect sizes close to 1.00 (IVW OR=1.00, 95% CI=0.87–1.22; MR-Egger OR=0.97, 95% CI=0.66–1.42; weighted median OR=0.96, 95% CI=0.71–1.28).

Our findings were corroborated by the results of two important sensitivity analyses. The leave-one-out analysis demonstrated the observed BMI-PCOS causal association (for both childhood and adult) was not driven by outlying variant. As shown in Additional file 2: Fig. S4, when iteratively removing one IV at a time and performing IVW using the remaining IVs, BMI-PCOS associations centered around an OR of 2.5–3.0, while WHR/WHR_adj_BMI-PCOS associations aggregated closely to 1.0. In the subsequent clustering analysis (Additional file 2: Fig. S5), for both childhood and adult BMI, we observed a clear linear trend in which BMI-increasing IVs also increased the risk of PCOS. On the contrary, no such pattern was observed for WHR or WHR_adj_BMI with PCOS, consistent with a null association.

**Fig. 4:**
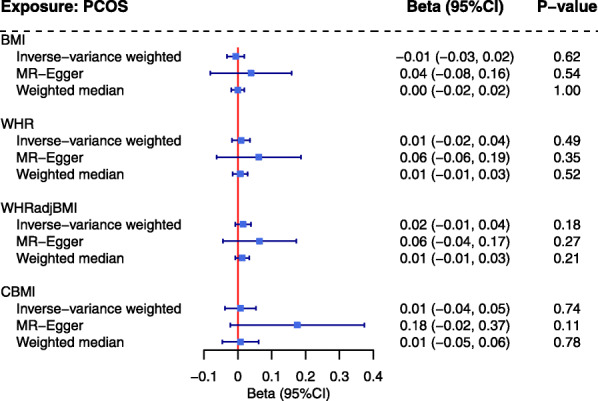
Estimates of causal effect sizes for genetical predisposition to PCOS on obesity-related traits using all genome-wide significant SNPs. Inverse-variance weighted approach was used as the primary analysis, MR-Egger and weighted median approaches were used as sensitivity analysis. BMI, adult body mass index; PCOS, polycystic ovary syndrome; WHR, waist-to-hip ratio; WHR_adj_BMI, waist-to-hip ratio adjusted for body mass index; CBMI, childhood body mass index

Genetic predisposition to PCOS did not seem to affect any of the obesity-related traits in our reverse-directional MR (adult BMI beta = − 0.01, 95% CI = − 0.03–0.02; childhood BMI beta = 0.01, 95% CI = − 0.04–0.05; WHR beta = 0.01, 95% CI = − 0.02–0.04; WHR_adj_BMI beta = 0.02, 95% CI = − 0.01–0.04) (Fig. 4).

## Discussion

To the best of our knowledge, this is the first large-scale genome-wide cross-trait analysis that investigates the shared genetic basis underlying obesity and PCOS. We found a positive overall genetic correlation between both adult and childhood BMI and PCOS. The significantly shared genetic basis between WHR and PCOS seems to be driven by BMI given the null finding of WHR_adj_BMI and PCOS. In local genetic correlation analysis, when the genome was partitioned into small regions, we identified one genomic region at chr18: 57630483–59020751 that showed a positive local genetic correlation between adult BMI and PCOS. Using cross-trait meta-analysis, we identified multiple shared loci between obesity and PCOS. Finally, MR analysis highlighted the causal role of adult BMI and childhood BMI in the development of PCOS.

Findings from our study are largely in line with those from the conventional epidemiological studies yet provide novel insights in several aspects. In line with the positive overall genetic correlation identified for adult BMI and PCOS (*r*_*g*_=0.34, *P*=8.21×10^−18^) by Day et al. [7], our results (adult BMI: *r*_*g*_=0.47, *P*=2.19×10^−16^) improved statistical power by using an enlarged sample size (434,794 female vs. 339,224 individuals) as well as reduced heterogeneity by using sex-specific adult BMI GWAS summary statistics, both of which previous studies did not have a chance for. Findings on the [41] overall and local genetic correlation between adult BMI and PCOS suggest a shared genetic basis underlying these two traits, which is either directly through variants affecting both traits (pleiotropy), or through the causal effect of one trait on the other. Our MR analyses exploring the causal relationships are in line with three existing MR(s) [7, 9, 10] conducted on BMI and PCOS while greatly extending those results. Firstly, we used the largest GWAS conducted in BMI with > 270 BMI-associated IVs—a more than three times augmented number of instruments (> 270 vs. 92) compared with previous analyses [7, 10]. Incorporating additional IVs substantially improves the strength of genetic instruments as well as both the accuracy and precision of our MR estimates. With the current sample size of outcome (N=113,238, 9% cases) and assuming phenotypic variance of the exposures explained by IVs to be around 4%, our study had 80% power to detect an association of 15% change for the risk of PCOS with BMI. Secondly, to ensure the validity of MR results, exposure and outcome samples should preferably be from the same underlying population. We conducted our analysis restricting to female participants—making it possible to utilize female-specific genetic instruments to study a female outcome PCOS, which previous analyses did not have the opportunity for. Finally, we conducted several sensitivity analyses to verify MR model assumptions. We selected the most significant SNPs (independent GWAS signals at a stringent P-threshold of 5×10^−9^) so all were robustly associated with exposure of interest, guaranteeing the “relevance” assumption. We excluded SNPs associated with potential confounders on the exposure-outcome relationship to satisfy the “exclusion restriction” assumption.

In addition to adult BMI, childhood BMI appears to influence the risk of PCOS later on [42, 43]. Findings from earlier observational studies including nearly 3000 participants from the Australian Childhood Determinants of Adult Health study (*N*=1516) and the biracial USA Babies substudy of the Bogalusa Heart Study (*N*=1247) have suggested an association between greater childhood BMI and PCOS among the white population (RR=4.05, 95% CI=1.10–14.83; RR=2.93, 95% CI=1.65–5.22) [43]. Our results of positive overall genetic, shared loci, and causal effect identified for childhood BMI with PCOS confirmed the observational association and provided genetic evidence for such association. For the null local genetic correlation, we note that the sample size of childhood BMI GWAS (*N*=39,620) is not sufficiently large for ρ-HESS recommendation (which 50,000 samples are preferably required). Future studies with larger sample sizes are warranted to replicate our findings.

Using WHR as a proxy to abdominal fat, we did not find any significant causal association for this trait with PCOS. Raw WHR is likely to be confounded by BMI as indicated by our previous genetic correlation analysis [29]. Dissecting the effect of BMI from WHR, the negative WHR_adj_BMI-PCOS causal association together with genetic correlation further confirmed the validity of our results. However, these results do not necessarily mean that abdominal visceral fat is not important in PCOS. While BMI is found to be highly correlated with excess fat mass (*r*=0.94) and abdominal visceral fat (*r*=0.71) [44], results for the association between fat distribution and PCOS from observational studies were contrasting. A study including 110 PCOS patients and 112 weight-matched controls assessed fat quantity and distribution using total-body dual x-ray absorptiometry concluded that women with PCOS had a higher quantity of central abdominal fat compared to weight-matched controls [45], however, recent imaging studies using a gold standard approach of magnetic resonance-based methodology demonstrated an equivalent visceral fat depot between women with PCOS and their BMI-/fat mass-matched controls [46–48]. Indeed, WHR poorly predicts the accumulation of visceral fat [49], which may explain our significant findings with BMI rather than with WHR. Moreover, studies have shown that waist circumference (WC) presents the highest correlation with magnetic resonance imaging measured abdominal visceral adiposity, the gold standard approach, as well as the best sensitivity and specificity in the receiver operating characteristic curve [50]—therefore is considered as a better indicator for abdominal obesity than WHR. Investigating WC and PCOS leveraging recently published female WC GWAS would be a future direction on this topic [51].

Genetic correlation provides genetic insights into the observational associations by estimating the degree of pleiotropy or causal overlaps shared by two traits while MR analysis infers causal relationships. We additionally performed a cross-trait meta-analysis to further dissect the complex genetic relationships between obesity and PCOS. Using cross-trait meta-analysis, we identified 16 SNPs shared between obesity-related traits and PCOS, indicating shared biological mechanisms underlying obesity and PCOS. Among these shared loci, we highlight the locus of *ERBB3*, *FTO*, *PROX1*, *GIPR*, and *MC4R* in relation to potential pathogenesis. *ERBB3* encodes epidermal growth factor receptors (EGFRs) and has been reported to be associated with PCOS by a meta-GWAS [7]. Studies have shown that gonadotropins upregulate *ERBB3* expression and EGFR signaling mediates LH-induced steroidogenesis, which plays an important role in follicular development [52]. EGFRs are also closely involved in obesity—experimental studies have shown that EGFRs are transactivated by leptin, a hormone of an elevated concentration in patients with obesity/metabolic syndrome [53]. For gene *FTO*, despite a large number of studies that have confirmed its contribution to adult obesity, childhood obesity, and obesity-related traits [36, 54–56], its role in PCOS remains controversial. Some studies have observed a positive association between *FTO* and PCOS whereas others have not [37, 57–59]. The association between *FTO* and PCOS may partially be mediated through obesity. Indeed, the most evident association between *FTO* and PCOS was observed in obese PCOS women, which was attenuated when adjusting for BMI [59]. Moreover, the impact of *FTO* on lipid oxidation in PCOS women might also contribute to the mechanism underlying the comorbidity of obesity and PCOS [60]. *PROX1* encodes one of the proteins of the homeobox transcription factor family, which plays an essential role in organ development during embryogenesis [61]. Results from both large-scale population study and mice models have shown that *PROX1* was associated with visceral fat accumulation [62, 63]. *PROX1* has also been found to be differently methylated in adipose tissue in PCOS women and controls [64]. In addition to its role in obesity, *PROX1* has also been linked to glycemic alteration (fasting glucose and type 2 diabetes) [38]—another important feature of PCOS. Similarly, *GIPR* influences glycemic traits including 2-h glucose level and insulin secretion [35]. *GIPR* encodes a G-protein coupled receptor for gastric inhibitory polypeptide (GIP), which has been demonstrated to stimulate insulin release in the presence of elevated glucose. Evidence has suggested that modulation of *GIPR* affects progesterone synthesis and expression of many progestogenic factors and enzymes that may involve in the subfertility feature of PCOS [65]. Notably, the shared region chr18: 57730096–57914679 (top SNP rs7228430) identified for childhood BMI and PCOS overlaps with the local genetic correlation identified for adult BMI and PCOS at chr18: 57630483–59020751, indicating shared biology underlying these traits. This region harbors *MC4R*, a gene previously found to be associated with higher adult BMI and childhood BMI. Using BMI-matched samples or BMI-adjusted statistical models, studies have found MC4R to be associated with an elevated BMI in PCOS [31, 66]. *MC4R* encodes melanocortin 4 receptor that plays an important role in central melanocortin neuronal pathways [67]. Furthermore, a study using PCOS rats models has also found an over-expression of the *MC4R* gene in the brain hypothalamus which may link to metabolic disorders [32].

Results from GTEx tissue enrichment analysis should be interpreted with caution due to the limited number of shared genes identified between obesity and PCOS. Similarly, the null findings from LDSC-SEG do not necessarily indicate a negligible shared biological mechanism given the limited sample size of PCOS GWAS and the positive findings identified by our other analyses.

We acknowledge several limitations. First, PCOS, as a complex disease, is classified into four phenotypes according to the presence or absence of ovulatory dysfunction, hyperandrogenism, and polycystic ovarian morphology [68]. Our study was not able to perform phenotype-specific analysis due to limited data availability. In addition, PCOS occurs in both obese/overweight and lean women, our findings of the role of obesity in PCOS may not be applicable to lean women with PCOS. A recent study using unsupervised clustering analysis suggested distinct genetic architecture underlying PCOS subtypes. Using biochemical and genotype data, PCOS can be classified into a “reproductive” subtype which presents higher luteinizing hormone (LH) and sex hormone-binding globulin (SHBG) levels with relatively low BMI, and a “metabolic” subtype which presents higher BMI, glucose, and insulin levels with lower SHBG and LH levels [69]. Our work was limited by the availability of subtype-specific PCOS GWAS, future work is needed to understand the role of BMI in lean PCOS. Second, the strong link between BMI and PCOS seems to be consistent across ethnicities, for example, using 78 BMI IVs discovered by a GWAS of Biobank Japan and 4,386 PCOS cases (8,017 controls) of East Asian ancestry, a twofold risk of PCOS was observed for heightened BMI (OR=2.21, 95% CI=1.54–3.17, *P*=1.8×10^−5^) [70], However, the generalizability of our findings of the shared genetic basis of obesity and PCOS is restricted to European population. Further genome-wide association studies on this topic leveraging data from other ethnicities are warranted. Third, despite our study being the (so far) largest in sample size, compared to adult obesity GWASs, PCOS has a much smaller sample size (4790 cases/20,405 controls vs. ~ 400,000 female), future studies with enlarged sample size are needed. Fourth, while our study identified genes relevant to obesity and PCOS, more data are needed to understand the underlying pathophysiological mechanisms.

Our work investigated the shared genetic basis underlying obesity and PCOS. Future work should be to perform large, prospective longitudinal clinical studies to define whether PCOS women carrying a specific genotype are at increased risk for developing, e.g., cardiovascular disease, type 2 diabetes, non-alcoholic fatty liver disease, and link to mortality. Of outmost importance, our study confirmed the causative role of BMI in PCOS prevention, future studies on whether medical weight reduction or bariatric surgery alleviates PCOS-related comorbidities are needed. Moreover, the use of gene-modified mice, e.g., knock-in/knock-out of obesity/PCOS risk genes is of importance to define the role of identified specific genes and rare genetic variants and would provide novel insights into the biological mechanisms underlying these complex traits.

## Conclusions

In conclusion, leveraging the largest sex-specific GWAS summary statistics to date, the current study furthered our understanding of the observational association between obesity and PCOS by showing evidence of genetic correlation, revealing shared loci, and inferring causal relationships, all of which may provide insights into the biological pathways. Our work informs public health intervention by confirming the important role of weight management from childhood through adulthood in PCOS prevention.

## Supplementary Information

**Additional file 1: Table S1.** Adult BMI IVs. **Table S2.** WHR IVs. **Table S3.** WHR_adj_BMI IVs. **Table S4.** childhood BMI IVs. **Table S50.** PCOS IVs. **Table S6.** Data sources, sample sizes, number of IVs and F-statistics. **Table S7.** Summary of contributing cohorts/consortia/studies in each GWAS. **Table S8.** Annotation of genome-wide significant SNPs from CPASSOC. **Table S9.** 99% credible set SNPs.

**Additional file 2: Figure S1.** Local genetic correlation between childhood BMI, WHR, WHR_adj_BMI and PCOS. **Figure S2.** GTEx tissue enrichment analysis. **Figure S3.** Cell-type specific enrichment analysis. **Figure S4.** Box plot of betas in leave-one-out analysis. **Figure S5.** MR-clust analysis.

## Data Availability

All data used in the present study were obtained from publicly available GWAS summary statistics.

## Abbreviations

BMI: Body mass index
CPASSOC: Cross-phenotype association analysis
DEG: Differentially expressed gene
EGFRs: Epidermal growth factor receptors
FUMA: Functional mapping and annotation
GWAS: Genome-wide associations studies
HRC: Haplotype Reference Consortium
IV: Instrumental variable
IVW: Inverse-variance weighted
LD: linkage disequilibrium
LDSC: linkage-disequilibrium score regression
LH: luteinizing hormone
MR: Mendelian randomization
PCOS: Polycystic ovary syndrome
PIP: Posterior inclusion probability
SHBG: Sex hormone-binding globulin
VEP: Variant effect predictor
WC: Waist circumference
WHR: Waist-to-hip ratio
WHR_adj_BMI: WHR adjusted for BMI
