# Supplementary material for: Genomic correlation, shared loci, and causal relationship between obesity and polycystic ovary syndrome: a large-scale genome-wide cross-trait analysis
Source: BMC Med. 2022 Feb 11;20:66. doi: 10.1186/s12916-022-02238-y (PMC8832782; doi:10.1186/s12916-022-02238-y)
Supplement: Supplementary file 2 — Additional file 2: Figure S1. Local genetic correlation between childhood BMI, WHR, WHRadjBMI and PCOS. Figure S2. GTEx tissue enrichment analysis. Figure S3. Cell-type specific enrichment analysis. Figure S4. Box plot of betas in leave-one-out analysis. Figure S5. MR-clust analysis. [file 12916_2022_2238_MOESM2_ESM.docx]

**
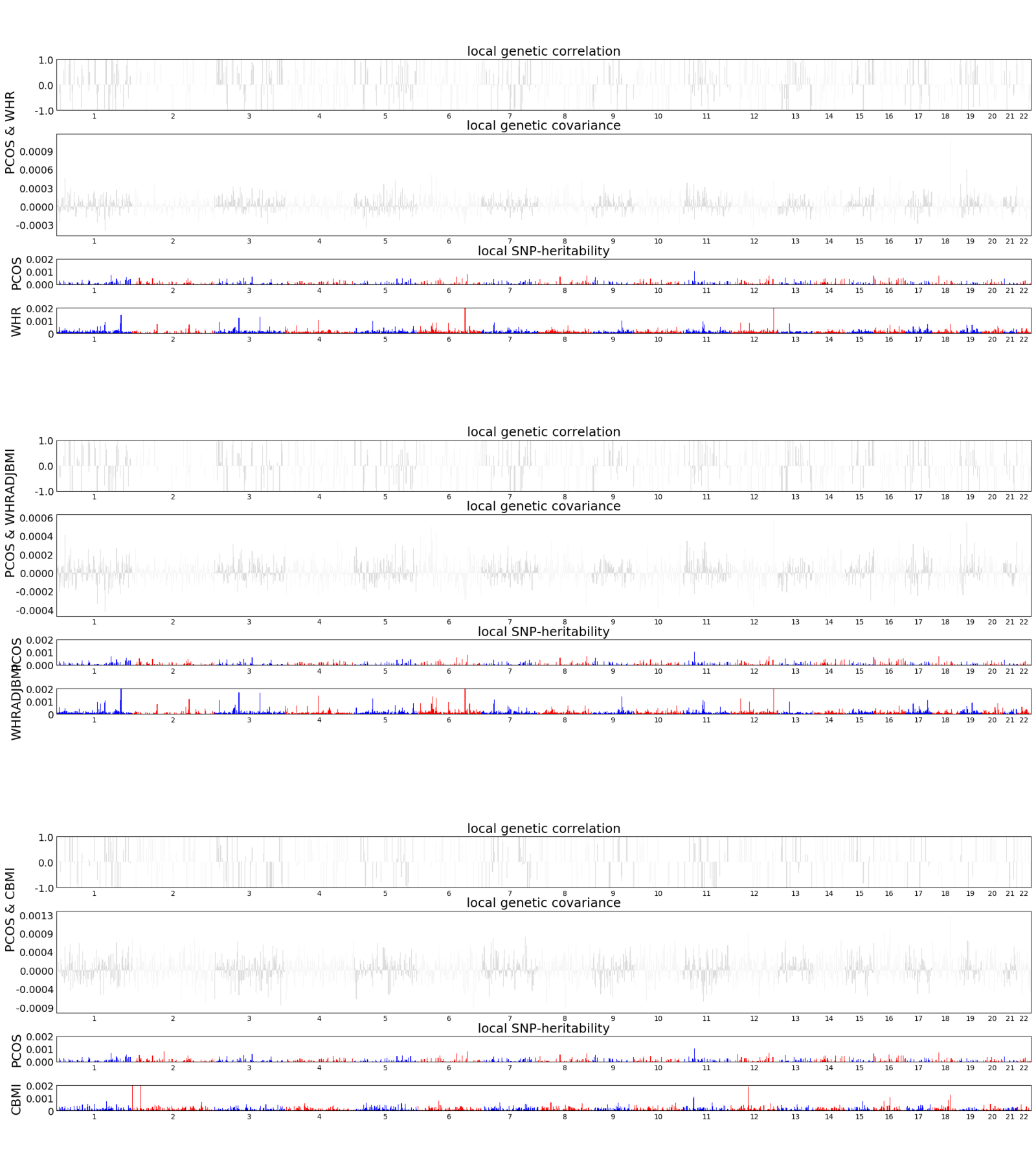
**

**Fig. S1.** Local genetic correlation, genetic covariance, and SNP heritability between childhood BMI, WHR, WHR_adj_BMI and PCOS. Colored bars represent loci that have significant local genetic correlation and covariance after multiple testing adjustment. WHR, waist-to-hip ratio; PCOS, polycystic ovary syndrome; WHR_adj_BMI, waist-to-hip ratio adjusted for body mass index; CBMI, childhood body mass index.


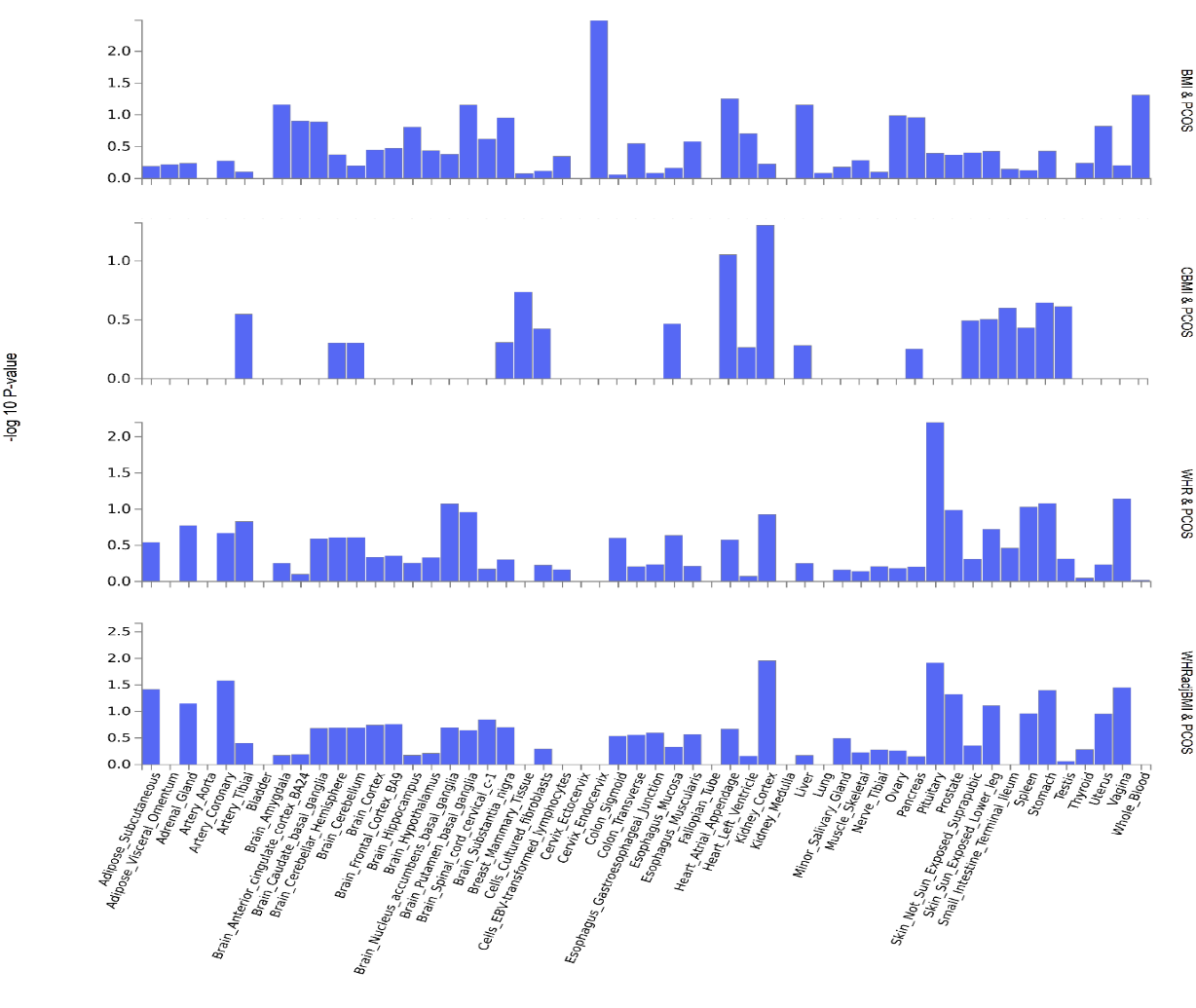


**Fig. S2.** GTEx tissue enrichment analysis for expression of all cross-phenotype associated genes within clumping area identified by CPASSOC for obesity-related traits and PCOS. Red represents significant tissue enrichment after Benjamin-Hochberg correction. BMI, adult body mass index; PCOS, polycystic ovary syndrome; WHR, waist-to-hip ratio; WHR_adj_BMI, waist-to-hip ratio adjusted for body mass index; CBMI, childhood body mass index.


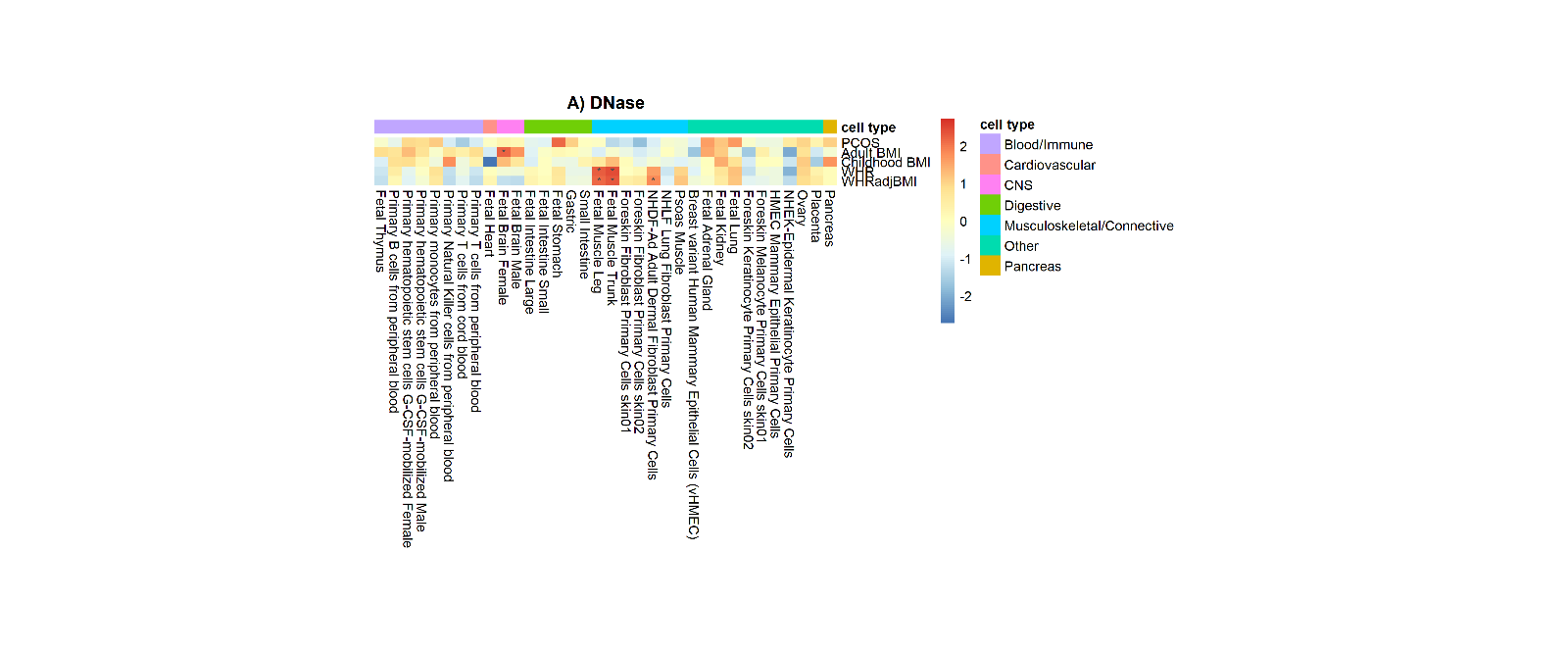

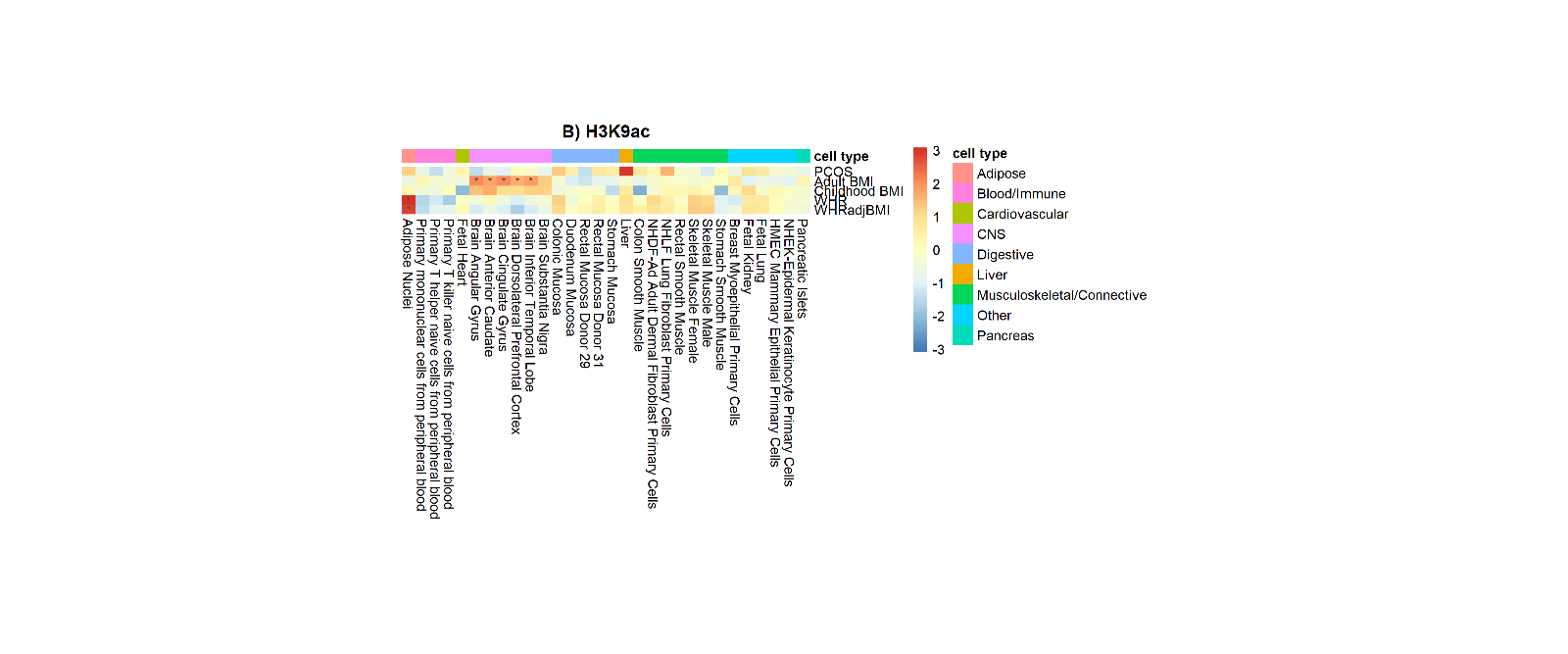

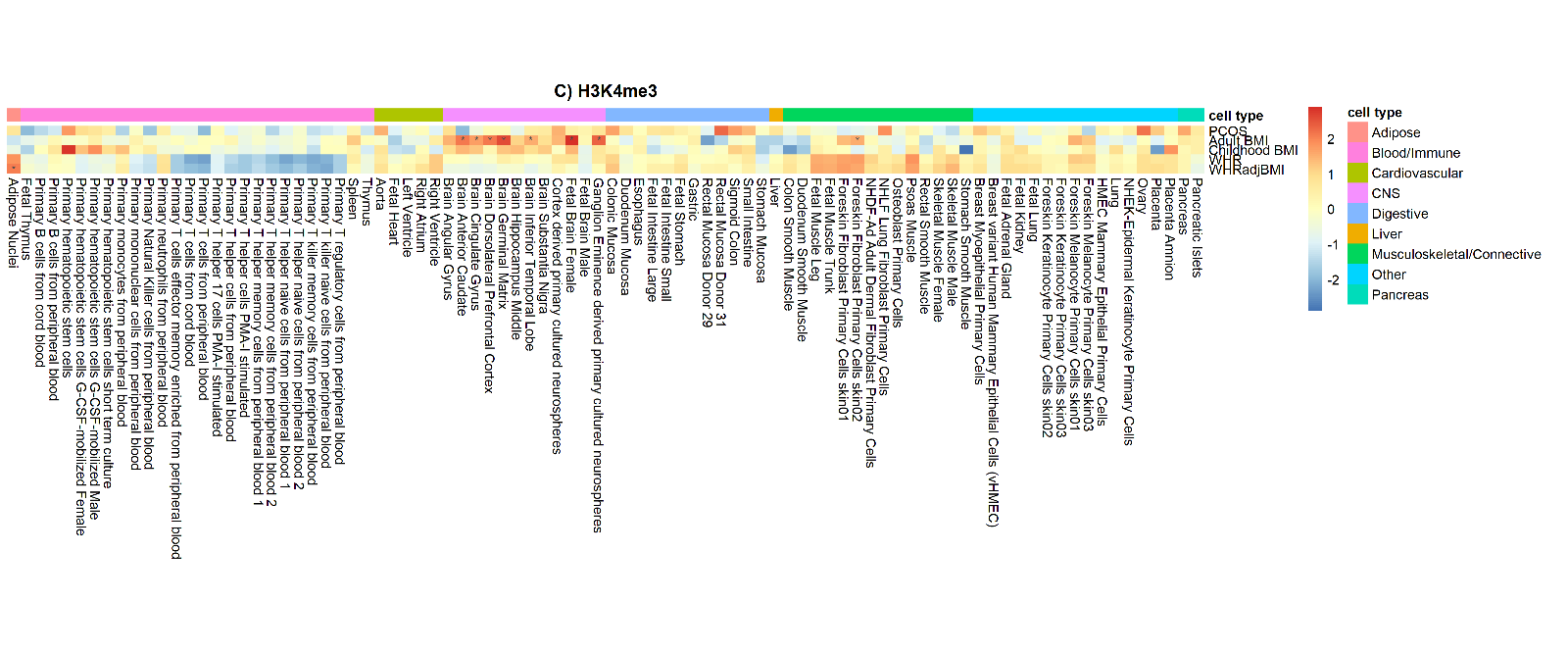

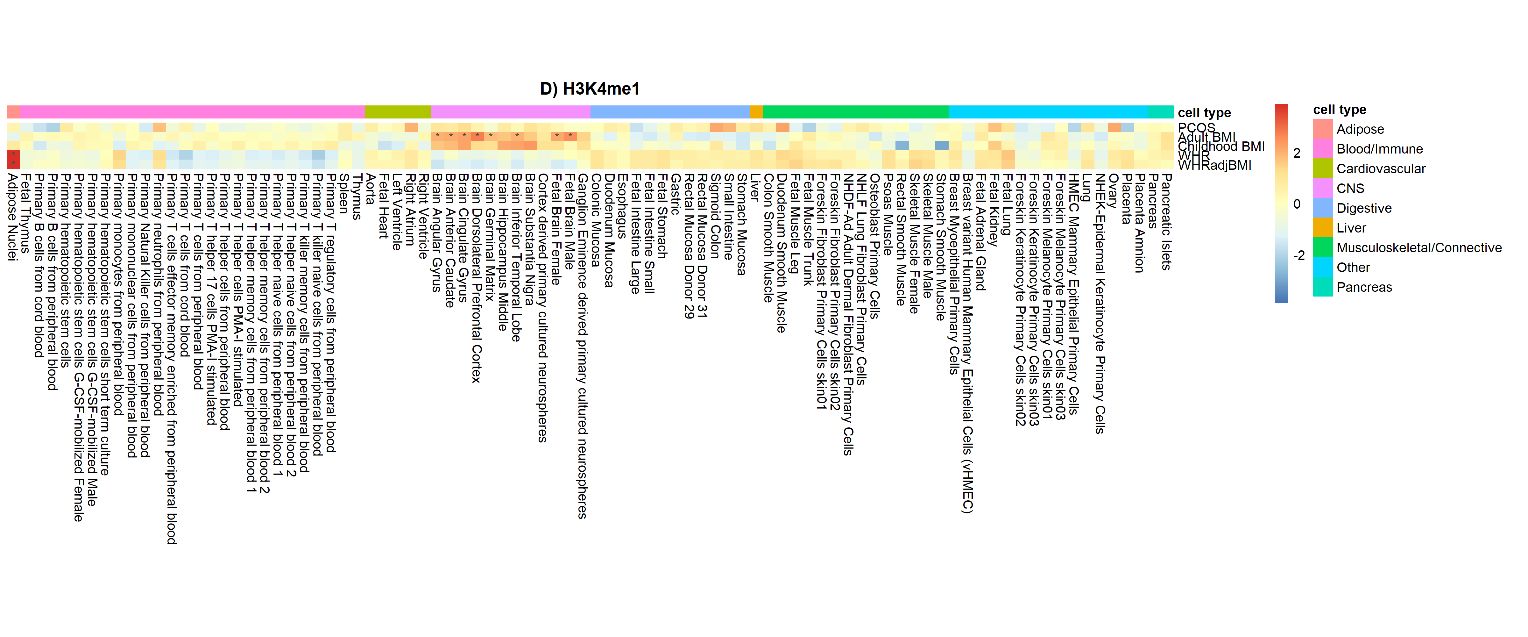

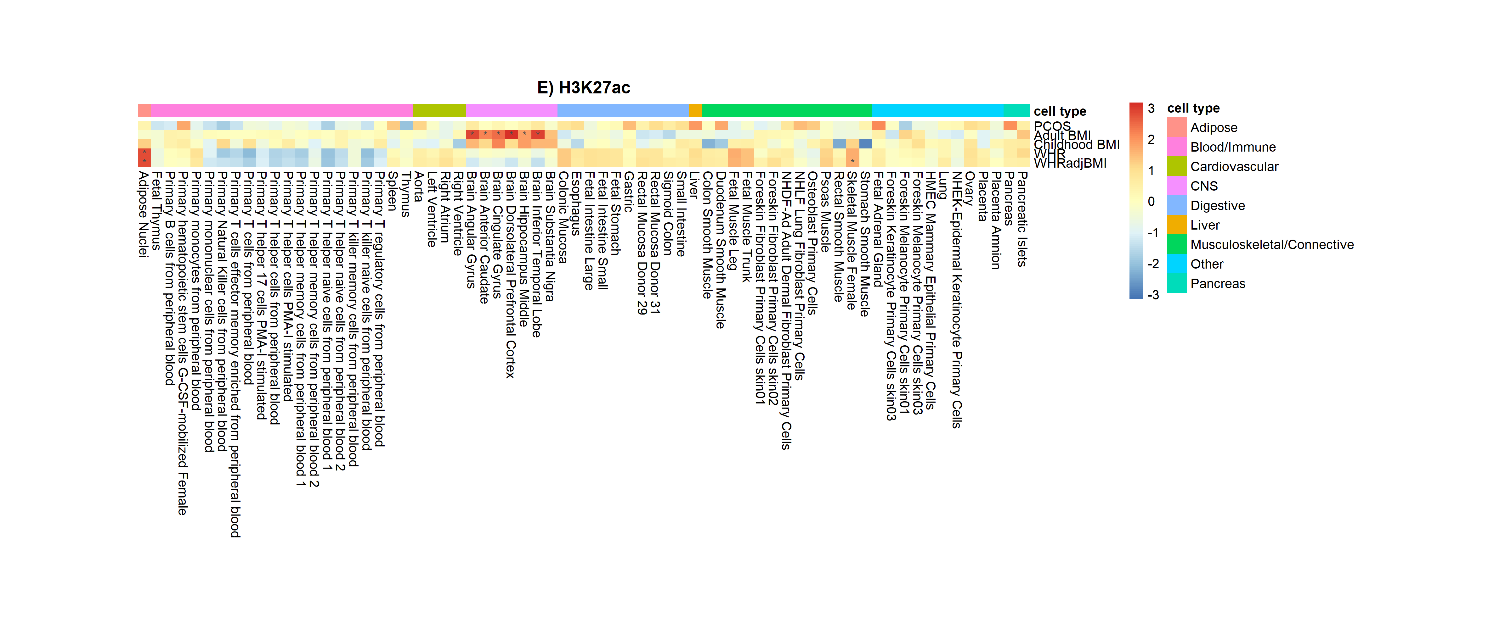

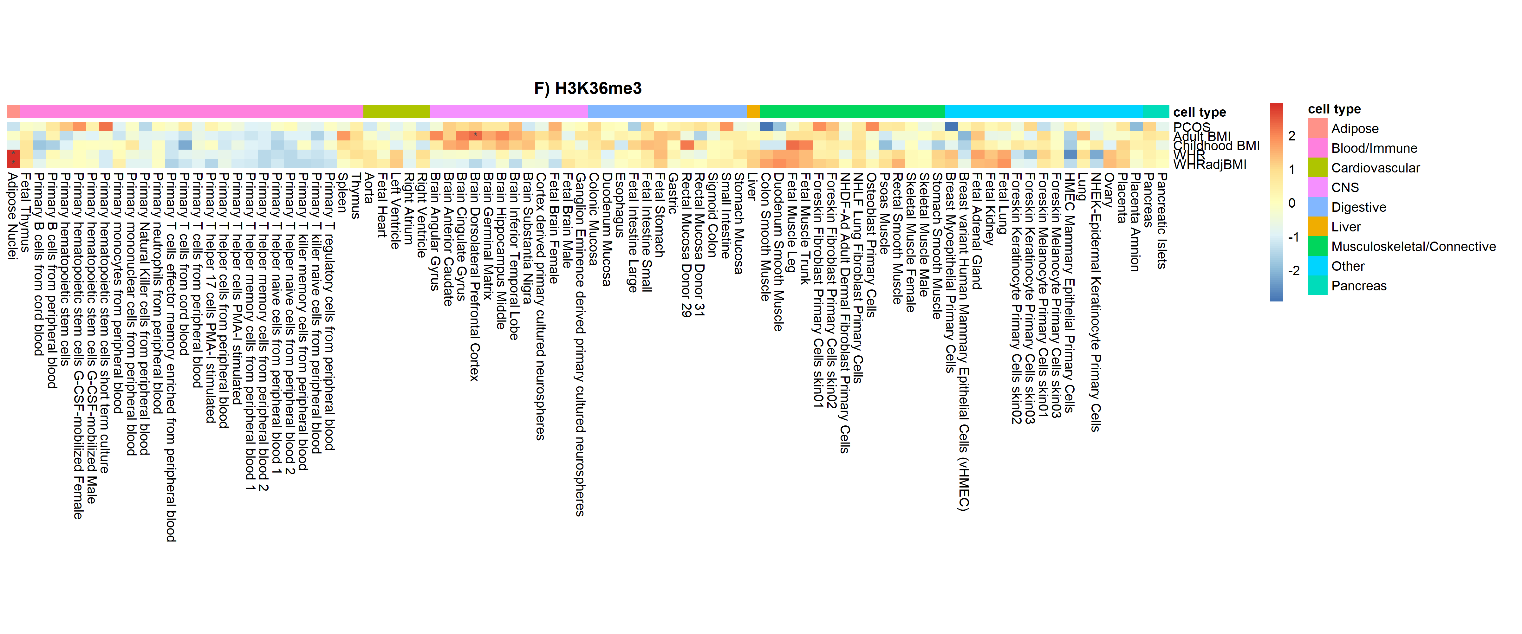


**Fig. S3.** Heatmaps of cell-type specific enrichment over six histone marks. We performed 396 tissues and cell-type-specific annotation analysis in PCOS as well as in four obesity-related traits. We compared these enrichment results using a heatmap. Each checker reflects the beta coefficient z-score, scaled by traits. Red indicates enrichment, blue indicates depletion. Deeper color represents a stronger magnitude of effects. Asterisk represents statistical significance withstood Bonferroni correction (*P*<0.05/396). The category of cell types is color-coded to the left. A) DNase, B) H3K9ac, C) H3K4me3, D) H3K4me1, E) H3K27ac and F) H3K34me3.

**
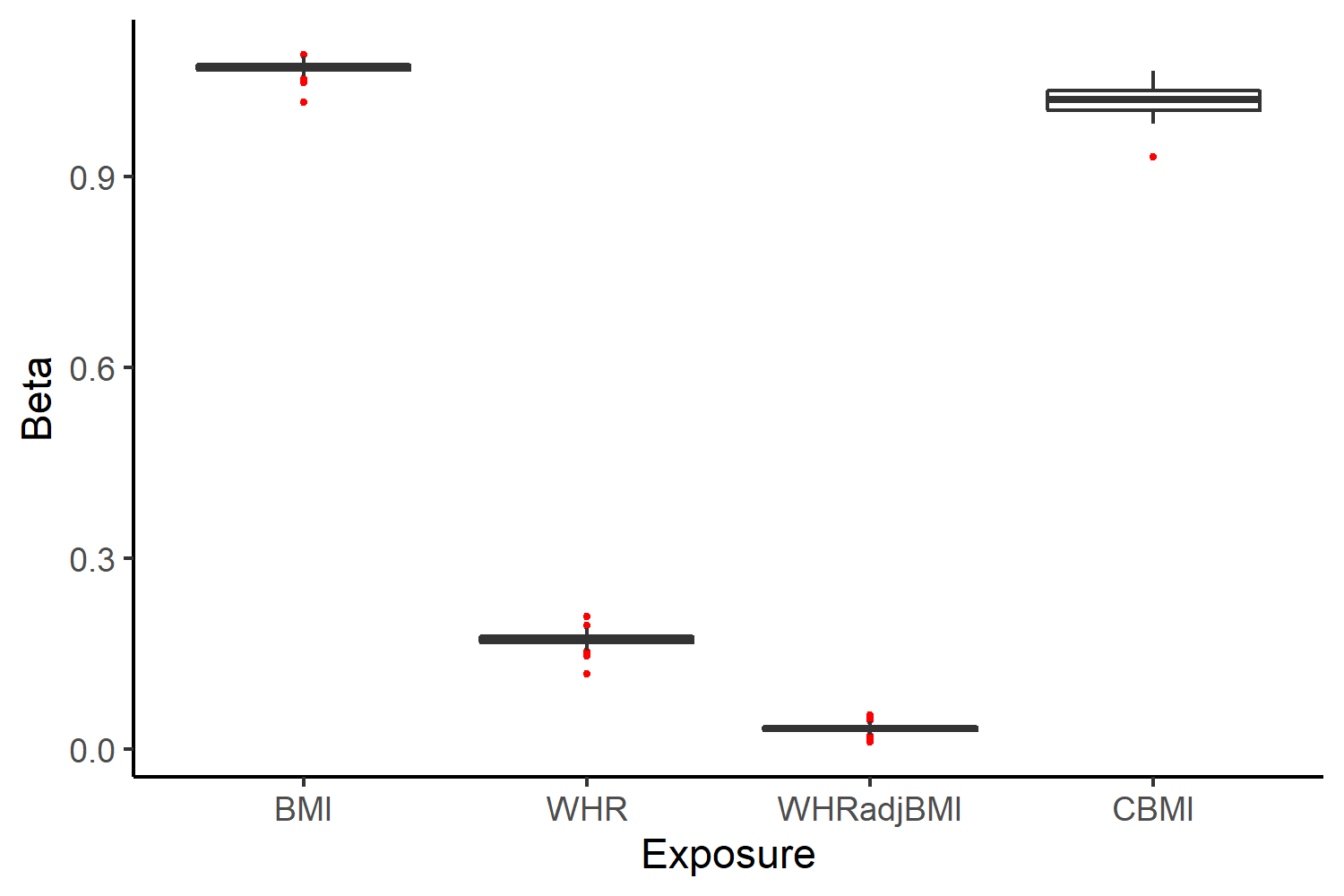
**

**Fig. S4.** Box plot of betas in leave-one-out analysis. Beta: effect allele beta coefficient; BMI: adult body mass index; WHR: waist-to-hip ratio; WHR_adj_BMI: waist-to-hip ratio adjusted for body mass index; CBMI: childhood body mass index.

**
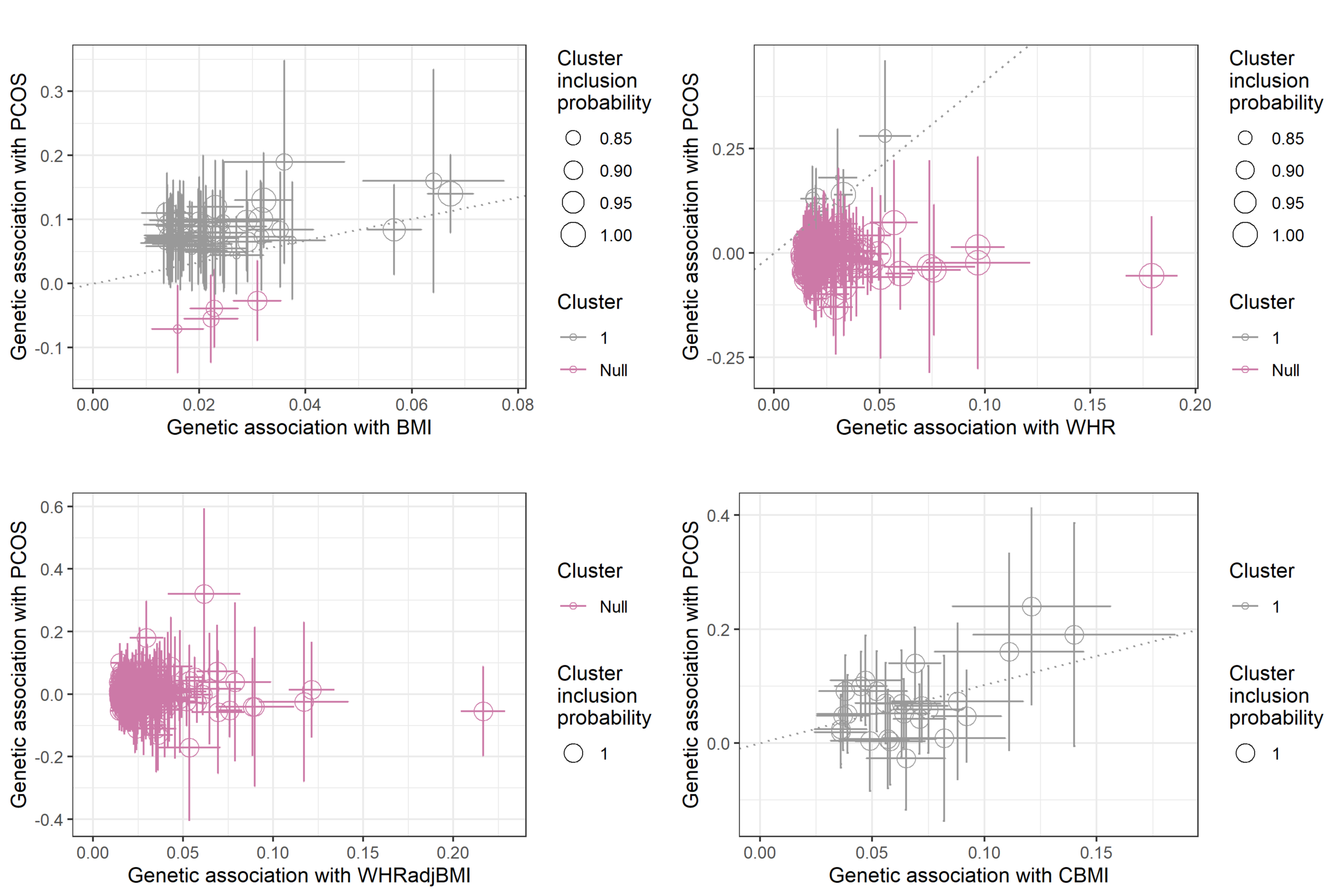
Fig. S5.** MR-clust analysis presenting genetic associations with obesity-related traits and PCOS disease risk (log odds) per additional obesity-related traits -increasing allele. Each point represents a SNP; error bars represent 95% confidence intervals for the genetic associations; colors represent the clusters; dotted lines represent the cluster means. SNPs are only assigned to a cluster if the conditional probability is ≥ 0.8⁠, and clusters are only displayed if at least 4 SNPs are assigned to the cluster. BMI: adult body mass index; WHR: waist-to-hip ratio; WHR_adj_BMI: waist-to-hip ratio adjusted for body mass index; CBMI: childhood body mass index; PCOS, polycystic ovary syndrome.
